# Towards a Field-Based Bayesian Evidence Inference from Nested Sampling Data

**DOI:** 10.3390/e26110930

**Published:** 2024-10-30

**Authors:** Margret Westerkamp, Jakob Roth, Philipp Frank, Will Handley, Torsten Enßlin

**Affiliations:** 1 Max Planck Institute for Astrophysics, 85748 Garching, Germany; roth@mpa-garching.mpg.de (J.R.); philipp@mpa-garching.mpg.de (P.F.); ensslin@mpa-garching.mpg.de (T.E.); 2 Faculty for Physics, Ludwig-Maximilians-Universität, 80539 Munich, Germany; 3 Department for Computer Science, Technical University Munich, 85748 Munich, Germany; 4 Cavendish Laboratory, Cambridge CB3 0HE, UK; wh260@cam.ac.uk; 5 Kavli Institute for Cosmology, Cambridge CB3 0EZ, UK

**Keywords:** nested sampling, information field theory, Bayesian inference, evidence calculation

## Abstract

Nested sampling (NS) is a stochastic method for computing the log-evidence of a Bayesian problem. It relies on stochastic estimates of prior volumes enclosed by likelihood contours, which limits the accuracy of the log-evidence calculation. We propose to transform the prior volume estimation into a Bayesian inference problem, which allows us to incorporate a smoothness assumption for likelihood–prior–volume relations. As a result, we aim to increase the accuracy of the volume estimates and thus improve the overall log-evidence calculation using NS. The method presented works as a post-processing step for NS and provides posterior samples of the likelihood–prior–volume relation, from which the log-evidence can be calculated. We demonstrate an implementation of the algorithm and compare its results with plain NS on two synthetic datasets for which the underlying evidence is known. We find a significant improvement in accuracy for runs with less than one hundred active samples in NS but a proneness for numerical problems beyond this point.

## 1. Introduction

In Bayesian inference, we update our knowledge about a measure of interest, which we call the signal, *s*, on the basis of some given data, *d*. This signal can be, among other things, a set of model parameters $\theta_{M}$, the model itself M, or a continuous field φ. Given the prior P(s), which describes our a priori knowledge about $s\in \{\theta_{M},M,\varphi ,...\}$, the likelihood P(d|s) of the data given the signal and the normalising constant P(d)=∫dsP(d|s)P(s), known as the evidence, Bayes’ theorem returns the posterior of the signal given the data,

(1) $$\begin{array}{c} P\left(s|d\right)=\frac{P(d|s)P(s)}{P(d)}. \end{array}$$

In particular, Bayesian parameter estimation is concerned with updating knowledge about a set of model parameters given the data. Here, the model parameters in general depend on the given model. Accordingly, the quantity of interest is the posterior $P(\theta_{M}|d,M)$. In the case of field inference, we aim to reconstruct a continuous field from a finite dataset by approximating the posterior probability P(φ|d). In fact, in both cases, Bayesian parameter estimation and Bayesian field inference, it is in general not necessary to compute the evidence, as long as one is interested in computing posterior expectation values. In contrast, in model comparison the evidence is the main measure of interest. In model comparison, multiple models, each with parameters and assumptions, are compared by the probability of a model $M_{i}$ given the data,

(2) $$\begin{array}{c} P\left(M_{i}|d\right)=\frac{P\left(d|M_{i}\right)P\left(M_{i}\right)}{P(d)}, \end{array}$$

with $P\left(d\right)=\sum_{j}P\left(d|M_{j}\right)P\left(M_{j}\right)$. Assuming that all models have the same a priori probability, this leads to a comparison of the evidences $P(d|M_{j})$ between a set of different models $\left\{M_{j}\right\}$. The ratio of the evidences of two models is called the Bayes factor, giving the betting odds for or against one of the models compared to the other.

A number of algorithms for Bayesian parameter estimation already exits, which can be divided into two classes of posterior estimation approaches. One approach approximates the posterior, i.e., it tries to find an analytic distribution that is close to the true posterior. The main approach here is variational inference (VI), which minimises a distance measure between the analytic distribution and the posterior distribution, for example the Kullback–Leibler divergence (KL) [1], and is often used in field inference. The other approach aims to generate a set of samples of the posterior distribution. This set of samples can be used to approximate the true posterior. The most popular posterior sampling algorithm is Markov Chain Monte Carlo (MCMC). A summary of the basics of MCMC and different implementations is given in [2]. MCMC methods draw samples directly from the posterior, given a likelihood and a prior model. The simplest algorithm for MCMC is the Metropolis–Hastings algorithm [3], which gives a biased random walk through the parameter space depending on a proposal function and the initialisation of the algorithm. Still, there are several challenges, such as the tuning of the proposal function and the initialisation, which lead to advanced MCMC methods such as ensemble sampling [4], Gibbs sampling [5] and Hamiltonian Monte Carlo [6].

For model comparison (Equation (2)), we are instead interested in the integration of the likelihood over the prior, or in other words in the evidence calculation. Computing Bayesian evidence is challenging in many applications, as discussed in [7]. The biggest challenges are high-dimensional posteriors with multiple, well-separated modes or plateaus and a high information gain from the prior to the posterior, increasing the amount of time the algorithm spends in the low posterior mass regime. A concise overview of the different approaches for integration is given in [8]. Two integration methods that smoothly contract the parameter space from the prior to the posterior are simulated annealing [9] and nested sampling (NS) [10,11]. While simulated annealing uses fractional powers of the likelihood to get from the prior to the posterior, NS instead takes samples from slices of the posterior and recombines them at the end. In particular, NS transforms the multidimensional problem of integrating the likelihood over the prior into a series of nested volumes defined by likelihood contours and the enclosed prior. In doing so, NS is able to estimate the log-evidence and the posterior samples simultaneously. The NS algorithm is analysed further from a physical perspective in [12].

As summarised in [13] there are several challenges in NS. First, the computational cost of NS depends on the choice of the prior, i.e., the broader the prior, the higher the computational cost. Second, sampling from the likelihood restricted prior is not trivial. Finally, the rate at which the posterior is integrated is a stochastic quantity for which there is only a probabilistic description. Accordingly, many improvements to NS have been proposed to address some of these challenges. A number of studies have focused on improving the calculation of evidence for likelihoods with peculiar shapes, such as likelihood plateaus. Likelihood plateaus violate the assumption of uniformity in sampling and lead to ambiguity in the ranking of samples, which often leads to an underestimation of the prior volume contraction and thus to an overestimation of the evidence. Accordingly, Ref. [14] proposed a preprocessing step to correctly handle the plateaus in the likelihood. The main idea is to decompose the parameter sets into disjoint subsets that divide into plateaus and parts on which the usual NS can be performed. Other studies have aimed to work with a variable rate at which the posterior is integrated, known as dynamic NS [15,16]. Dynamic NS is particularly useful for parameter estimation, as standard NS spends a great deal of computational effort navigating to the posterior peak. Ultimately, dynamic NS allows more samples to be placed in regions where we want higher resolution, However, most of the progress has been focused on improving the sampling process for the likelihood restricted prior sampling (LRPS). There are generally two different approaches that focus on LRPS - rejection sampling, used by MultiNest [17], and chain-based sampling using Markov chains, implemented for example in PolyChord [18]. In addition, Ref. [19] noted that standard NS assumes independent prior samples given the likelihood constraint. However, this is usually not the case, leading to a bias in the evidence calculation. Accordingly, they introduce nested sampling via sequential Monte Carlo (NS-SMC) [20] based on the idea of importance sampling, which does not require the imprecise assumption of independent samples.

The dominant error in the evidence calculation, which is based on the statistical estimate of the shrinkage ratio $t_{i}$, can be reduced by taking a larger number of samples, which is where improvements in LRPS focus. We take an orthogonal approach and try to increase the accuracy of NS through a post-processing step that reduces the statistical error in each of the compression factors. To do this, we use information field theory (IFT) to perform Bayesian field inference to reconstruct a continuous and smooth likelihood–prior–volume function given the likelihood contour information from NS. The presented approach has been addressed in [21]. In this paper, we aim to give a deeper introduction into the post-processing and perform further validation.

In the following, we first give a general overview of the methods used in Section 2. This includes an introduction to NS and its notation, an introduction to IFT and the general explanation of one-dimensional correlated field inference in Section 2.2, and finally the method for inferring prior volume estimates and a possible implementation of it in Section 2.3. In Section 3, we show the according inference results for two validation examples. In particular, we choose a Gaussian likelihood and a spike-and-slab likelihood [22]. Finally, we discuss the results, including an analysis of the computational cost, and conclude in Section 4.

## 2. Methods

### 2.1. Nested Sampling Algorithm

Using the notation introduced by [10] the NS likelihood is denoted via L(θ):=P(d|θ) and the prior is denoted by π(θ):=P(θ). The evidence, Z:=P(d) is calculated accordingly,

(3) $$\begin{array}{c} Z=\int d\theta L(\theta )\pi (\theta ). \end{array}$$

The idea of NS is to transform this possibly high-dimensional integral in parameter space into a one-dimensional one. Given the prior mass, *X*, enclosed by some likelihood contour L(θ)=L,

(4) $$\begin{array}{c} X\left(L\right)=\int_{L(\theta )>L}d\theta \;\pi \left(\theta \right), \end{array}$$

we can rewrite Equation (3) to a one-dimensional integral,

(5) $$\begin{array}{c} Z=\int_{0}^{1}dX\;L\left(X\right), \end{array}$$

where L(X) is the likelihood value on the θ-contour that encloses the prior mass *X* (Equation (4)). The underlying algorithm for the calculation of the integral in Equation (5) can be summarised as follows: First, $n_{\mathrm{live}}$ samples are drawn from the prior π(θ), which we call the live points, $\theta_{1},...,\theta_{n_{\mathrm{live}}}$. For each of these samples the likelihood can be calculated, $d_{L,i}=L\left(\theta_{i}\right)$. Then, the sample *j* out of these with the lowest likelihood is added to a new set, called the dead points, ${\vec{d}}_{L}:=\left\{d_{L,j}\right\}$. A new sample is drawn, restricted to the space of higher likelihood values. This is called likelihood restricted prior sampling (LRPS). Accordingly, we transfer samples from a set of live points to dead points with increasing likelihood while adding new samples to the set of live points for which the likelihood values exceed the highest dead contour. This leads to the condition $d_{L,i}>d_{L,i-1}$. The prior volume under consideration shrinks at each iteration, $X_{i}<X_{i-1}$, by a compression factor $t_{i}$,

(6) $$\begin{array}{c} X_{i+1}=t_{i}X_{i}. \end{array}$$

Under the assumption that the samples are drawn from the prior independently within the highest dead contour, all compression factors, $\vec{t}=\left\{t_{i}\right\}$, are independent of each other and beta-distributed, $P\left(t_{i}\right)=\mathrm{Beta}\left(t_{i}|1,n_{\mathrm{live}}\right)$. The algorithm stops after $n_{\mathrm{iter}}$ iterations. Finally, the set of dead points and estimated prior volumes, defined by Equation (6), are used to approximate the evidence using the quadrature rule with the according weights $\omega_{i}$,

(7) $$\begin{array}{c} Z\approx Z=\sum_{i=1}^{n_{\mathrm{iter}}}\omega_{i}d_{L,i}. \end{array}$$

In this study, we use weights defined by the trapezoidal rule $\omega_{i}=\frac{1}{2}\left(X_{i-1}-X_{i+1}\right)$ with $X_{0}=1$ and $X_{n_{\mathrm{iter}}+1}=0$.

**Figure 1:**
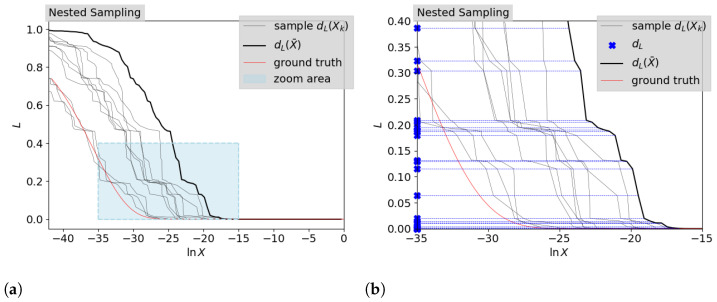
Illustration of nested sampling (NS) output for simple Gaussian example introduced in [11] and further elaborated in Section 3.1 with two live points. (**a**) shows the full NS data generated with anesthetic and (**b**) shows a zoomed-in section, which is indicated in (**a**). The zoomed-in image additionally shows the information of the likelihood dead contours ${\vec{d}}_{L}$, which we use as data for the Bayesian inference of the prior volumes. In both figures, the samples of likelihood–prior–volume functions defined by prior volume samples, $X_{k}$, ${\vec{d}}_{L}\left(X_{k}\right)$, are shown as well as a likelihood–prior–volume function defined by the deterministic NS approach in Equation (8), ${\vec{d}}_{L}\left(\bar{X}\right)$.

In view of this procedure, NS introduces a statistical uncertainty, since the prior volumes at each iteration, $X_{i}$, are not known, but only the distribution of the contraction factors defining the prior volumes is known. In the literature, there are two different approaches for the estimation of the prior volume mentioned. The first one, which we call the statistical approach, samples *K* chains of compression factors ${\left\{t_{i}\right\}}_{k}$ independently. Correspondingly, we can define several sets of prior volumes ${\left\{X_{i}\right\}}_{k}$, where for each chain k=1,...,N the prior volume at iteration *i* is defined by the corresponding sets of contraction factors, $X_{i,k}=\prod_{j=1}^{i}t_{j,k}$. The result is *K* samples for the log-evidence using Equation (7), which allows us to obtain the mean estimate of the log-evidence and its uncertainty. The second approach, which we call the deterministic approach, instead gives no uncertainty estimate. Here, the mean of the log-compression factors ${\langle \ln t_{i}\rangle }_{P(t_{i})}=-1/n_{\mathrm{live},i}$ is taken as an estimate, as discussed in [23]. This yields the deterministic prior volume estimation,

(8) $$\begin{array}{c} {\bar{X}}_{i}=\prod_{j=1}^{i}{\langle t_{j}\rangle }_{P(t_{j})}=e^{\ln(\prod_{j=1}^{i}{\langle t_{j}\rangle }_{P(t_{j})})}\approx e^{\sum_{j=1}^{i}-1/n_{\mathrm{live},j}}, \end{array}$$

and hence ${\bar{X}}_{i}\approx e^{-i/n_{\mathrm{live}}}$ if the number of live points remains constant at each iteration [18]. In other words, the prior volume gets compressed exponentially. In Figure 1, we show the likelihood–prior–volume curves generated by NS for a simple Gaussian example, which was introduced by [11]. In Section 3.1, the details of this simple Gaussian case are discussed further. The according NS likelihood contours were generated using the software package anesthetic [24]. Figure 1a shows the entire likelihood–prior–volume function generated together with the analytical ground truth. Figure 1b shows an enlarged section that is marked in the left panel. In NS each likelihood dead contour, $d_{L,i}$, is accompanied by the estimated prior volume, described by all contraction factors up to the considered iteration. This leads to the corresponding NS likelihood–prior–volume function, defined through either the statistical prior volume estimation, ${\vec{d}}_{L}\left(X_{k}\right)$ for k=1,...,N, or the deterministic prior volume estimation, ${\vec{d}}_{L}\left(\bar{X}\right)$. Both panels of Figure 1 show both the statistical and the deterministic likelihood–prior–volume function. The zoomed-in panel also shows the information on the likelihood contours given by NS, which we will use as the only data, ${\vec{d}}_{L}$ for the inference of the likelihood–prior–volume function.

Despite NS being mainly designed to estimate the evidence, it generates posterior samples and their individual probabilities, $p_{i}$, at each iteration $i=1,...,n_{\mathrm{iter}}$ of the model parameters $\theta_{M}$ as a by-product as described in [18],

(9) $$\begin{array}{c} p_{i}=\frac{\omega_{i}d_{L,i}}{Z}=\frac{\omega_{i}d_{L,i}}{\sum_{i}\omega_{i}d_{L,i}}. \end{array}$$

These posterior samples can be used to compute the posterior expectation values of any function of the model parameters $g(\theta_{M})$,

(10) $$\begin{array}{c} {\langle g\left(\theta_{M}\right)\rangle }_{P(\theta_{M}|d)}=\int d\theta_{M}g\left(\theta_{M}\right)P\left(\theta_{M}|d\right)\approx \frac{1}{n_{\mathrm{iter}}}\sum_{i=1}^{n_{\mathrm{iter}}}p_{i}g\left(\theta_{i}\right). \end{array}$$

One such function and specific quantity of interest is the information gain from the prior to the posterior given by the KL,

(11) $$\begin{array}{c} H:=D_{\mathrm{KL}}\left(P\left(\theta |d\right)|\pi \left(\theta \right)\right)=\int d\theta P\left(\theta |d\right)\ln\left(\frac{P(\theta |d)}{\pi (\theta )}\right)=\int_{0}^{1}dX\frac{L(X)}{Z}\ln\left(\frac{L(X)}{Z}\right). \end{array}$$

The information gain is an important quantity for estimating the number of steps $n_{\mathrm{iter}}$ needed to reach the posterior mass. It is therefore a good measure for determining a termination criterion. In particular, as noted in [10,25], the posterior set is reached after about $n_{\mathrm{live}}H$ steps and the posterior is passed in $n_{\mathrm{live}}\sqrt{C}$ steps, where *C* is the number of dimensions. That is, more live points $n_{\mathrm{live}}$ provide better sampling of the posterior, but also increase the time to reach the posterior, increasing the overall computation. In addition to the number of live points, $n_{\mathrm{live}}$, the information gain *H* and the average computational cost for LRPS at each iteration and for evaluating the likelihood have an impact on the total computational cost T. In particular, T scales as $O(n_{\mathrm{live}})$ and $O(H^{2})$ as pointed out in [26]. Looking at the error ϵ for the evidence calculation using NS, we find that, according to [27], it is composed of three components under the assumption that we integrate up to the $n_{\mathrm{iter}}$th iteration using Equation (7),

(12) $$\begin{array}{cc} \epsilon & =\sum_{i=1}^{n_{\mathrm{iter}}}\omega_{i}d_{L,i}-\int_{0}^{1}L\left(X\right)dX\\ \; & =\underset{\mathrm{truncation}\;\mathrm{error}}{\underset{︸}{-\int_{0}^{X_{n_{\mathrm{iter}}}}L\left(X\right)dX}}+\underset{\mathrm{numerical}\;\mathrm{integration}\;\mathrm{error}}{\underset{︸}{\left(\sum_{i=1}^{n_{\mathrm{iter}}}\omega_{i}L\left(X_{i}\right)-\int_{X_{n_{\mathrm{iter}}}}^{1}L\left(X\right)dX\right)}}+\underset{\mathrm{stochastic}\;\mathrm{error}}{\underset{︸}{\sum_{i=1}^{n_{\mathrm{iter}}}\omega_{i}\left(d_{L,i}-L\left(X_{i}\right)\right)}}. \end{array}$$

According to [16], the numerical integration error introduced by replacing the integral by the trapezoidal rule is of the order of $O(1/n_{\mathrm{live}}^{2})$ and therefore negligible as the number of live points goes to infinity. The truncation error occurs when we stop at a given maximum iteration $n_{\mathrm{iter}}$. It can be kept small by choosing the stopping criterion wisely. Several approaches have been described in the literature to determine the final iteration $n_{\mathrm{iter}}$, ranging from simultaneously computing the information *H* to determine the location of the posterior set [11], to stopping as soon as the LRPS becomes inefficient [28] or as soon as the expected evidence from the remaining live points compared to the current evidence estimate is less than a user-defined tolerance [16,18,23].

The error we are most interested in and aim to minimise is the stochastic error introduced by the unknown prior volumes. This error is of the order of $O(n_{\mathrm{live}}^{-\frac{1}{2}})$ [27], or as derived in [10], proportional to $\sqrt{H/n_{\mathrm{live}}}$, and dominates the evidence approximation error. As a result, the stochastic error trends to zero as the number of live points goes to infinity, but at the same moment the computational cost trends to infinity. Thus, there is a trade-off between the accuracy of the evidence calculation and the computation time. In the following, we will present a post-processing step for NS that aims to reduce either the error in the evidence calculation or the time complexity, depending on the measure of interest.

### 2.2. Information Field Theory

We use IFT [29] for the joint reconstruction of the continuous likelihood–prior–volume function and the discrete set of prior volumes. IFT focuses on Bayesian field inference, or in other words, on the reconstruction of a continuous field from a discrete dataset. Here, we consider the likelihood–prior–volume function to be a one-dimensional field with an infinite number of degrees of freedom, which is to be reconstructed from a finite set of likelihood dead contours, ${\vec{d}}_{L}$. Thus, the inference problem is underconstrained, and we need prior knowledge of the likelihood–prior–volume function and the prior volumes to obtain the posterior. We call this prior probability distribution the joint reconstruction prior $P(L\left(X\right),\vec{t})$ to avoid confusion with the prior volumes of NS. The reconstruction likelihood $P({\vec{d}}_{L}|L\left(X\right),\vec{t})$ is then the probability of the measured likelihood dead contours given the likelihood–prior–volume function. We combine the information on the reconstruction prior and the reconstruction likelihood in Bayes’ theorem (Equation (1)) to reconstruct the posterior probability of the field L(X), which we call the reconstruction posterior $P(L\left(X\right),\vec{t}|{\vec{d}}_{L})$. This allows us to obtain any a posteriori measure of interest, like for example the mean and variance of the likelihood–prior–volume function. In Section 2.3, we will introduce the explicit reconstruction likelihood and prior models for the here introduced inference. Here, we focus on using IFT and its software package Numerical Information Field Theory (NIFTy) [30] to implement a generative prior for the likelihood–prior–volume function, considering its correlation structure.

Since the prior model is based on Gaussian processes, this section describes Gaussian processes from the IFT perspective. Specifically, we introduce a generative model for Gaussian processes with variable correlation structure. In other words, the aim is to generate a field, τ, as a Gaussian process G(τ,T) with an unknown covariance *T*. This implementation, which is desirable in many cases, ensures the smoothness of the likelihood–prior–volume function. We use this information in the presented algorithm to improve the accuracy of the evidence calculation. In the following sections, we will discuss the smoothness assumption and its positive and negative consequences in more detail. One approach for the implementation of a non-parametric model for fields with unknown correlation structure, was introduced in [31]. Analogously, we call this model the correlated field model, which suggests that not only the realisation of the field itself, τ, is learned, but also the underlying correlation structure *T*. Here, we consider the simplest case of a one-dimensional correlated field. The correlated field is implemented as a generative process using the reparametrisation trick introduced in [32], $\tau =A\xi_{\tau }$ with $T=AA^{†}$ and $P\left(\xi_{\tau }\right)=G\left(\xi_{\tau },I\right)$. We can separate the field realisation from the field correlation structure using this basis transformation. This means that the new coordinates, $\xi_{\tau }$, have the same dimension as τ, but are a priori uncorrelated. Assuming statistical homogeneity and isotropy, the covariance *T* is fully defined by its power spectrum $p_{T}\left(|k|\right)$ via the Wiener–Khinchin theorem in Fourier space [33],

(13) $$\begin{array}{c} A_{kk^{\prime }}={\left(FAF^{†}\right)}_{kk^{\prime }}=2\pi \delta \left(k-k^{\prime }\right)\sqrt{p_{T}\left(|k|\right)}, \end{array}$$

where *F* is the Fourier transform and $\sqrt{p_{T}\left(|k|\right)}$ is the amplitude spectrum. The aim is to infer the power spectrum non-parametrically. This is achieved by building a model of the power spectrum where each hyperparameter is described by a Gaussian or log-normal prior with a given mean and standard deviation. Each hyperparameter, and thus the entire power spectrum, is learned during inference. More specifically, the amplitude spectrum is implemented as an integrated Wiener process, a general continuous process, on the logarithmic scale l=log(|k|) for k≠0,

(14) $$\begin{array}{c} \sqrt{p_{T}\left(l\right)}\propto e^{\gamma (l)},\frac{d^{2}\gamma }{dl^{2}}=\eta \xi_{W}\left(l\right),P\left(\xi_{W}\right)=G\left(\xi_{W},I\right). \end{array}$$

The integration gives

(15) $$\begin{array}{c} \gamma \left(l\right)=ml+\eta \int_{l_{0}}^{l}\int_{l_{0}}^{l^{\prime }}\xi_{W}\left(l^{\prime \prime }\right)dl^{\prime }dl^{\prime \prime }. \end{array}$$

Here, $l_{0}$ is the first mode greater than zero and *m* defines the slope of the integrated Wiener process, i.e., it is the slope of the amplitude spectrum on a double logarithmic scale. The parameter η is called flexibility because it controls the total variance of the integrated Wiener process. In addition to these parameters, *m* and η, which essentially determine the shape of the power spectrum, the total offset of the correlated field defined by the zero mode and another hyperparameter, called the fluctuations, *a*, which specifies the total fluctuations of the non-zero modes,

(16) $$\begin{array}{c} \sqrt{p_{T}\left(l\right)}=a\frac{e^{\gamma (l)}}{{\left(\int_{l_{0}}^{l}e^{2\gamma (l^{\prime })}dl^{\prime }\right)}^{\frac{1}{2}}}, \end{array}$$

are introduced. The expression in the denominator normalises, so that the meaning of *a* as the fluctuation amplitude is invariant under the change of γ, which determines on which Fourier scales the fluctuations appear. Overall, this gives a generative model for a Gaussian random field with unknown covariance.

**Figure 2:**
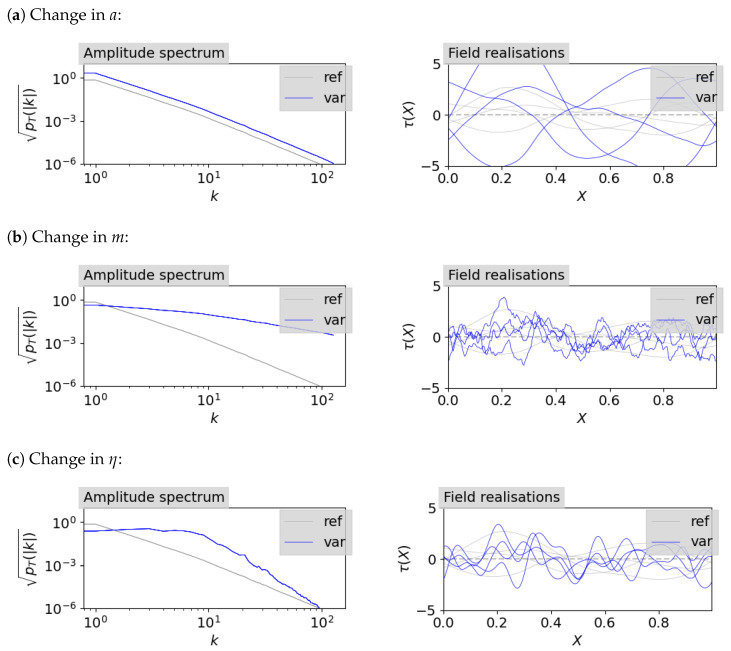
Visualisation of the effect of changes in the mean value for one of the hyperparameters of the power spectrum model. The changed amplitude spectrum is shown on the left side and the according influence on the field realisation is shown on the right side by comparison of a reference correlated field (ref) and a variation of one of its hyperparameters (var). Which hyperparameter is changed and how it is changed in comparison to the reference is denoted in Table 1.

The effect of changing *a*, *m*, and η is shown in Figure 2. It shows a reference power spectrum and corresponding sample field realisations, together with the power spectrum and field realisations for a changed mean of one of the hyperparameters. The variances of the hyperparameters are the same for all cases and are kept small in order to better show the effect of the hyperparameters on the correlated field. The specific means and variances of the hyperparameters are listed in Table 1. For the reconstruction itself, we keep the prior wide, which means that we take higher values for the standard deviations of each parameter to allow for flexibility of the model.

IFT performs Bayesian field inference to infer the posterior for a continuous field given some data $d_{\tau }$. The exact relationship between the correlated field, τ, and the likelihood–prior–volume function is discussed in Section 2.3. Thereby, the posterior probability $P(\tau |d_{\tau })$ is approximated by a simpler posterior distribution $Q(\tau |d_{\tau })$ using variational inference (VI). The approximation is done by minimising the cross-entropy term of the KL between the actual posterior and its approximation $D_{\mathrm{KL}}\left(Q\left(\tau |d_{\tau }\right)|P\left(\tau |d_{\tau }\right)\right)$. In particular, we use the geometric variational inference (geoVI) introduced by [34]. The geoVI algorithm optimises the cross-entropy of the KL with respect to a non-linear normalising coordinate transformation that maps the posterior onto a standard Gaussian. This allows it to approximate non-Gaussian posteriors. All numerics related to IFT are implemented in the corresponding software package NIFTy [30].

### 2.3. Bayesian Inference of the Likelihood–Prior–Volume Function

As noted in [21], we use the smoothness assumption for the likelihood–prior–volume curve to improve the evidence calculation in NS. The description of the algorithm is given below using the simple Gaussian example introduced by [11] for illustration. The full information from NS for this case is presented in Figure 1a. As mentioned in Section 2.1, NS generates data on the likelihood dead contours ${\vec{d}}_{L}$. These data points are marked in Figure 1b on the likelihood axis. However, to compute the evidence we would need the likelihood–prior–volume function, including information on the prior volumes. In the following, we aim to give an algorithm that aims to improve the overall estimate on the prior volume and thereby reduces the uncertainty in the evidence.

**Figure 3:**
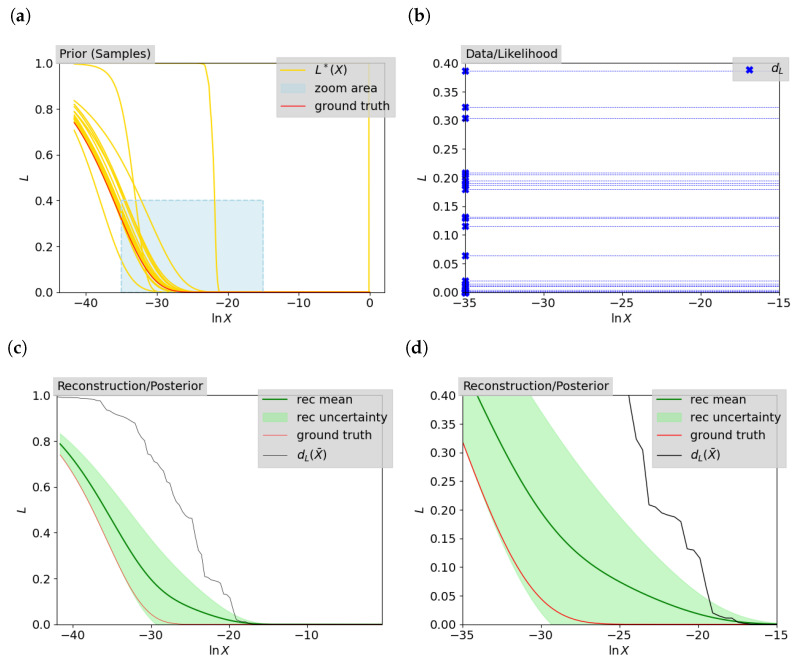
Illustration of the Bayesian field inference process for the simple Gaussian, which is further elaborated in Section 3.1 for two live points. The prior samples, the data used, and the final reconstruction compared to the NS approach and the ground truth are shown. (**a**): Prior samples for the likelihood–prior–volume function ($L^{*}\left(X\right)$) in yellow together with the ground truth. A zoomed-in area is marked beside it (the same area as in Figure 1a) which is taken to zoom into the data in (**b**) and the reconstruction in (**d**). (**b**): Data on likelihood dead contours for the given zoom area. (**c**,**d**): Reconstruction mean (rec mean) of the likelihood–prior–volume function and the associated uncertainty, defined via the one sigma contours (rec uncertainty), which is zoomed in on in (**d**), and the full image is presented in Figure (**c**). Moreover, the result for the likelihood–prior–volume function for the deterministic NS approach is shown ($d_{L}\left(\bar{X}\right)$), which is the same as in Figure 1b, and the analytic likelihood–prior–volume-function (ground truth).

We propose to jointly infer the likelihood–prior–volume function L(X) and the prior volumes $\left\{X_{i}\right\}$ at each iteration $i=1,...,n_{\mathrm{iter}}$ using Bayesian field inference as described in Section 2.2. For the data, we only take into account the information we obtain from NS about the probability of dead contours, ${\vec{d}}_{L}$. The composition of the reconstruction is shown in Figure 3a. As ingredients for Bayes theorem, we use the joint reconstruction prior model for the contraction factors and the likelihood–prior–volume function, as well as the reconstruction likelihood model, which in our case is defined fully by the data. Figure 3a shows prior samples for the likelihood–prior–volume function described by the correlated field to be learned. The data obtained by NS for the likelihood dead contours, which then describes the reconstruction likelihood, are shown in Figure 3b. Merging these two models and approximating the reconstruction posterior with VI yields posterior samples of the likelihood–prior–volume function and the set of prior volumes. Figure 3c shows the computed mean and uncertainty for the reconstructed posterior likelihood–prior–volume function, as well as the function obtained by pure NS and the analytic ground truth. In Figure 3d, a zoomed area on the reconstructed likelihood–prior–volume function is shown, in order to facilitate comparison with Figure 1b.

Below, we describe a method to enforce the smoothness assumption on the likelihood–prior–volume curve using the correlated field model introduced in Section 2.2. In Appendix C, we present an alternative approach that requires no δ-function approximation; however, this method is currently only applicable for a MAP estimate and not for VI. The here presented approach implements the smoothness by describing the derivative of the likelihood by the prior volume as a log-normal process, which can be achieved by using a correlated field as described in Section 2.2. By using a log-normal rather than a Gaussian process for the derivative of the logarithmic likelihood–prior–volume relation, we ensure that the likelihood–prior–volume function is monotonic. As a consequence of the correlated field model described above, it would be desirable that,

(17) $$\begin{array}{c} -e^{-\tau (\ln X)}=\frac{d\ln L}{d\ln X}\approx \mathrm{const}, \end{array}$$

with τ drawn from a Gaussian process. However, it can be seen that for the simplest case, a Gaussian likelihood model, this assumption is not fulfilled. Accordingly, we introduce a reparametrisation *f* that maps lnL such that we find a damped log-normal process for $\frac{d\ln L}{d\ln X}$,

(18) $$\begin{array}{c} \frac{df_{\ln L}}{d\ln X}:=\frac{df(\ln L)}{d\ln X}=\frac{1}{\ln L_{\max}-\ln L}\frac{d\ln L}{d\ln X}=-e^{-\tau (\ln X)}. \end{array}$$

This way, a constant τ perfectly captures the Gaussian case, and non-Gaussianity is absorbed in excitations of τ around this constant, as described in Section 2.2. The derivation of the reparametrisation for the Gaussian case is given in Appendix A. Appendix B shows how to calculate the corresponding $L_{\max}$ according to [35] if it is not known analytically. The joint reconstruction prior for the reparametrised likelihood–prior–volume function $f_{\ln L}$ and the contraction factors $\vec{t}$ is fully defined via the joint prior $P(\tau ,\vec{t})$,

(19) $$\begin{array}{c} P\left(f_{\ln L},\vec{t}\right)=P\left(f_{\ln L}|\vec{t}\right)P\left(\vec{t}\right)=G\left(\tau ,T\right)\prod_{i=1}^{n_{\mathrm{iter}}}\mathrm{Beta}\left(t_{i}|1,n_{\mathrm{live},i}\right)=P\left(\tau ,\vec{t}\right). \end{array}$$

The reconstruction likelihood is described by the solution of the differential equation in Equation (18), which can be written as a function of the set of contraction factors,

(20) $$\begin{array}{c} f_{\ln L}\left(\vec{t}\right)=f_{\ln L}\left(\ln X_{j}=\sum_{i=1}^{j}t_{i}\right)=f_{\ln L}\left(0\right)-\int_{0}^{\sum_{i=1}^{j}t_{i}}e^{-\tau (z)}dz. \end{array}$$

This leads to a likelihood model, which is a δ-function, which we approximate by a Gaussian with a small chosen variance $\sigma_{\delta }$,

(21) $$\begin{array}{c} P\left(f\left(\ln{\vec{d}}_{L}\right)|\tau ,\vec{t}\right)=\delta \left(f\left(\ln{\vec{d}}_{L}\right)-f_{\ln L}\left(\vec{t}\right)\right)\approx G\left(f\left(\ln{\vec{d}}_{L}\right)-f_{\ln L}\left(\vec{t}\right),\sigma_{\delta }\right). \end{array}$$

The result is the joint reconstruction posterior,

(22) $$\begin{array}{c} P\left(\tau ,\vec{t}|f\left(\ln{\vec{d}}_{L}\right)\right)\propto P\left(f\left(\ln{\vec{d}}_{L}\right)|\tau ,\vec{t}\right)P\left(\tau ,\vec{t}\right). \end{array}$$

The posterior is approximated using geoVI as described in Section 2.2, slowly increasing the number of samples from iteration to iteration. We choose $\sigma_{\delta }=0.1\;\min\left(\mathrm{dist}\left(f\left(\ln{\vec{d}}_{L}\right)\right)\right)$ to ensure that the likelihood does not allow for the exchange of two data points or even non-monotonicity. Finally, the prior of τ prefers a constant flat τ without excitation, corresponding to a linear relation between $f_{\ln L}$ and lnX, corresponding to a Gaussian likelihood in NS. However, the prior of $\vec{t}$ prefers certain distances between the prior volumes given by the beta distribution. The likelihood ensures that the reconstructed function $f_{\ln L}$ evaluated at the reconstructed prior volumes *X* matches the values of $f(\ln{\vec{d}}_{L})$. This means that, for example, if there is a jump in $f(\ln{\vec{d}}_{L})$, P(τ) prefers a corresponding jump in lnX to ensure smoothness and to avoid deviations from the linear reparametrised likelihood–prior–volume relation, while $P(\vec{t})$ on average prefers an increase in lnX defined by the beta distribution.

## 3. Results and Analysis

For validation, we consider two cases: a simple Gaussian case as described in [11] and a spike-and-slab likelihood as introduced in [22]. The according data on the likelihood live and dead contours are generated using the anesthetic package by [24]. These test cases are valuable for checking the consistency of the presented method, as they allow the analytical calculation of evidence.

### 3.1. Gaussian Case

As a first validation test case, we use a zero-centred Gaussian likelihood,

(23) $$\begin{array}{c} L\left(\theta \right)=\exp\left(-\frac{r^{2}}{2\sigma^{2}}\right)\;\;\;\mathrm{with}\;\;\;r^{2}=\sum_{i=1}^{C}\theta_{i}^{2},\;\;\theta ={\left\{\theta_{i}\right\}}_{i=1,...,C}, \end{array}$$

in C=10 dimensions with as variance σ=0.02. As a prior, we use, in analogy to [11], a flat prior on the unit sphere

(24) $$\begin{array}{c} \pi \left(\theta \right)=\frac{C/2!}{\pi^{C/2}}\;\;\;\mathrm{with}\;\;\;r<1. \end{array}$$

The evidence given the probability and prior above can be calculated analytically to be

(25) $$\begin{array}{c} Z=\int_{-\infty }^{\infty }d\theta^{C}\;L\left(\theta \right)\pi \left(\theta \right)=\frac{(C/2)!}{{\left(2\sigma^{2}\right)}^{C/2}}. \end{array}$$

The definition of the prior mass in *C* dimensions is given by $X=r^{C}$, which allows us to compute the ground truth of the likelihood–prior–volume function,

(26) $$\begin{array}{c} L\left(X\right)=\exp\left(\frac{-X^{2/C}}{2\sigma^{2}}\right). \end{array}$$

**Figure 4:**
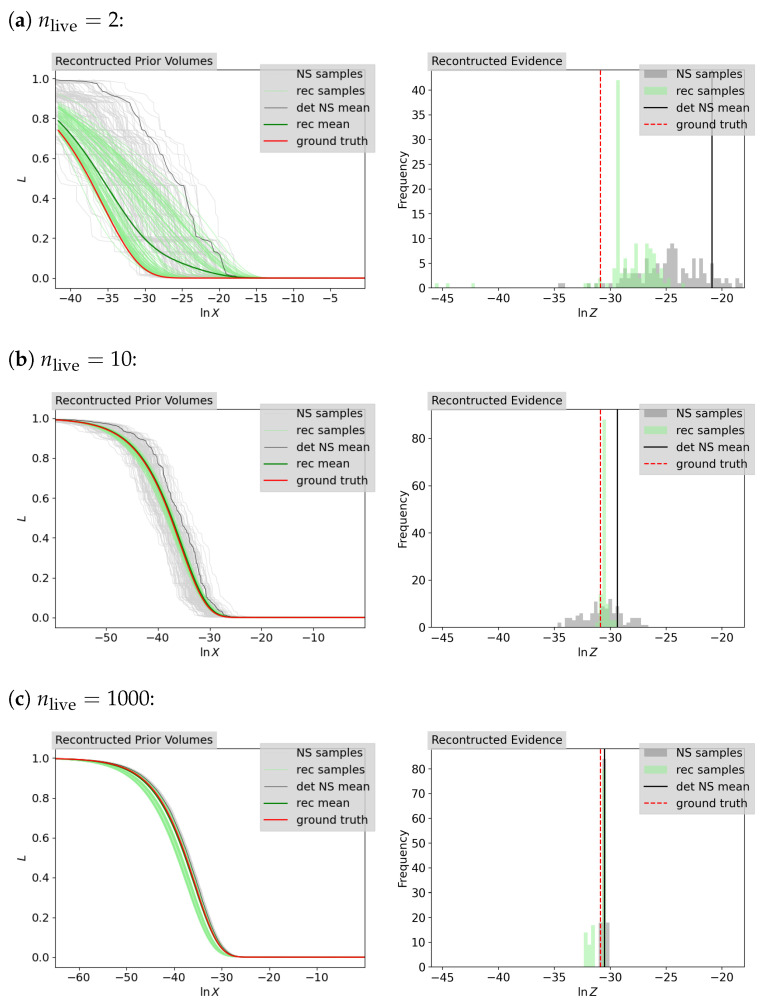
Reconstruction results for the Gaussian prior volumes accompanied by the likelihood contours given by NS on the left and the computed log-evidence on the right for $n_{\mathrm{live}}\in \left\{2,10,1000\right\}$ from top to bottom. The inferred posterior samples (rec samples) are shown together with their mean (rec mean) and compared with the corresponding statistical (NS samples) and deterministic (det NS mean) NS results and the ground truth.

Figure 4 shows the ground-truth likelihood–prior–volume function together with samples of the likelihood–prior–volume function defined by the reconstructed prior volumes or the statistical approximated prior volumes from NS for a constant number of live points, $n_{\mathrm{live}}\in \left\{2,10,1000\right\}$. Also shown is the likelihood–prior–volume function for the deterministic NS approach using Equation (4). As described in Section 2.1, it can be seen that the standard deviation for the NS approach decreases as the number of live points increases. For each of these prior volume estimation approaches, the statistical NS, the deterministic NS or the IFT based, we calculate the log-evidence using the weighted sum in Equation (7). This gives us sample sets of evidence for the statistical NS and IFT approaches, which are plotted as a histogram in Figure 4 together with the analytical ground truth and the deterministic NS approach. The corresponding results for each of the approaches for the mean and, where applicable, the standard deviation are given in Table 2. Further discussion of the results can be found in Section 4.

### 3.2. Spike-and-Slab Case

As a next step we consider a non-Gaussian test case for validation. In particular, we will look at a spike-and-slab likelihood known from Bayesian variable selection. Ref. [22], which is the sum of a zero-centred spike and a broad Gaussian background. This leads to an abrupt change in the prior volume *X* with increasing likelihood. The corresponding likelihood is given by

(27) $$\begin{array}{c} L\left(\theta \right)=a\exp\left(-\frac{r^{2}}{2\sigma_{1}^{2}}\right)+\left(1-a\right)\exp\left(-\frac{r^{2}}{2\sigma_{2}^{2}}\right)\;\;\;\mathrm{with}\;\;\;r^{2}=\sum_{i=1}^{C}\theta_{i}^{2}. \end{array}$$

Again, we choose a flat prior as described in Equation (24). Accordingly, we are able to calculate the evidence analytically, which gives us a good point for comparison,

(28) $$\begin{array}{c} Z=\int_{-\infty }^{\infty }d\theta^{C}L\left(\theta \right)\pi \left(\theta \right)=C/2!\left(a{\left(2\sigma_{1}^{2}\right)}^{C/2}+\left(1-a\right){\left(2\sigma_{2}^{2}\right)}^{C/2}\right). \end{array}$$

Just as in Section 3.1, we can obtain the analytic likelihood–prior–volume function given the prior volume $X=r^{C}$,

(29) $$\begin{array}{c} L\left(X\right)=a\exp\left(\frac{-X^{2/C}}{2\sigma_{1}^{2}}\right)+\left(1-a\right)\exp\left(\frac{-X^{2/C}}{2\sigma_{2}^{2}}\right). \end{array}$$

The parameters of the spike-and-slab likelihood under consideration are denoted in Table 3.

**Figure 5:**
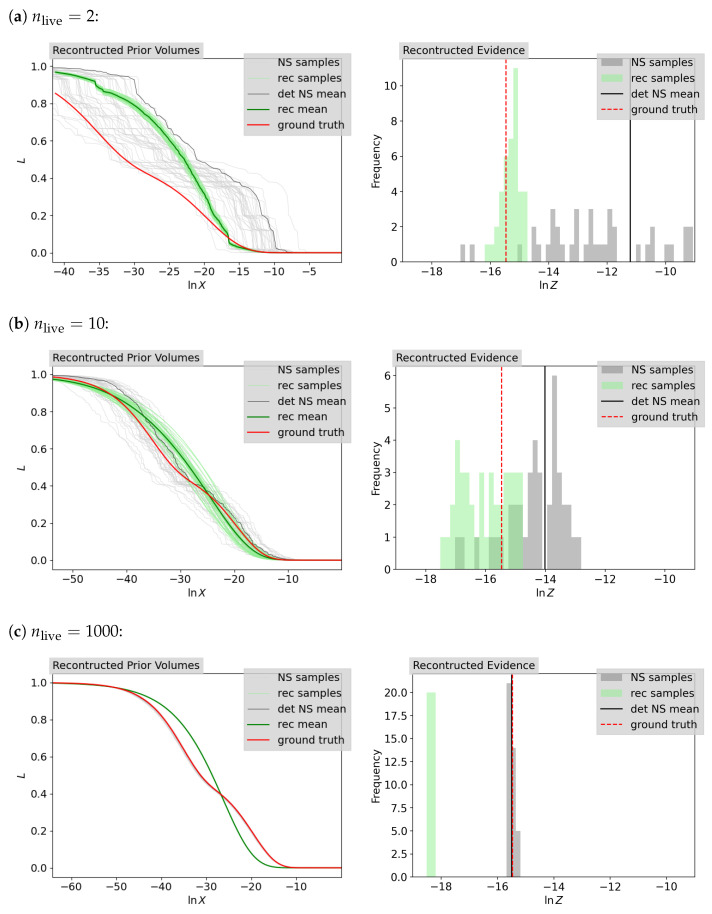
Reconstruction results for the spike-and-slab prior volumes accompanied by the likelihood contours given by NS on the left and the computed log-evidence on the right for $n_{\mathrm{live}}\in \left\{2,10,1000\right\}$ from top to bottom. The inferred posterior samples (rec samples) are shown together with their mean (rec mean) and compared with the corresponding statistical (NS samples) and deterministic (det NS mean) NS results and the ground truth.

Figure 5 shows the ground truth of the likelihood–prior–volume function (Equation (29)) and evidence (Equation (28)) together with the corresponding samples and mean (Equation (4)) given by the corresponding NS runs for $n_{\mathrm{live}}\in \left\{2,10,1000\right\}$. We infer the likelihood–prior–volume function and the set of prior volumes jointly using the inference algorithm described in Section 2.3. As a result, we obtain a set of posterior samples on the likelihood–prior–volume functions and the prior volumes, which leads to a set of posterior samples of the evidence using Equation (3). The posterior samples for the likelihood contours as a function of the reconstructed prior volumes (rec samples) as well as their mean (rec mean) are shown on the left side of Figure 5. The computed corresponding evidence is shown on the right side of Figure 5. For comparison, additionally the results for the statistical NS approach and the deterministic NS approach as well as the ground truth are shown. The computed mean evidences for the sample sets for the IFT and the NS approach are listed in Table 4 together with the corresponding standard deviation. A further discussion of the results is given in Section 4.

## 4. Discussion

In NS, the statistical error in one of the contraction factors $t_{i}$ affects each upcoming prior volume according to Equation (6). For this reason, it has a major impact on the calculation of the logarithmic evidence. However, the propagation of error does not occur on the likelihood contour information, which is assumed to be accurate, but only on the prior volume estimates. We use additional knowledge to be able to assign an improved estimate of the prior volume to the corresponding likelihood contour. The assumption we take into account is the smoothness of the likelihood–prior–volume function, which is valid for a large set of problems. Of course, if a problem is considered where the likelihood–prior–volume function is not smooth, this post-processing algorithm will not be applicable and the inference of the prior volumes could even make the result worse. One extreme example, which was discussed in [36], is the wedding cake likelihood. Furthermore, the inference algorithm has its limits when dealing with likelihood plateaus. The reason for this limitation is that we model the derivative of the likelihood–prior–volume function as a modified log-normal process. A zero slope would therefore correspond to an infinite excitation of the correlated field τ. A way to deal with this problem was suggested by [14]. Accordingly, one could split the dataset into several parts and perform the inference solely on the strictly negative monotonic regions of the likelihood–prior–volume functions. The same applies to the use of nested sampling to compute the evidence for multimodal likelihoods. In [37], three different approaches to LRPS for multimodal likelihoods are presented; as long as the final likelihood–prior–volume function would be given by a smooth function that could be partitioned around possible plateaus, the algorithm outlined here is well-suited for the task.

In terms of computational cost, our overall goal is to define a post-processing algorithm whose computational cost is independent of the number of live points, $n_{\mathrm{live}}$. As described in Section 2.1, the computational cost of a NS run is proportional to the number of live points $n_{\mathrm{live}}$ and the KL, *H*, while the error in lnZ is proportional to $\sqrt{H}$ and anti-proportional to $\sqrt{n_{\mathrm{live}}}$. Therefore, a large shrinkage from prior to posterior increases both the error and the computation time, while the error can be reduced by using more live points, leading to an increase in computation time. Accordingly, if the inference described here reduces the error by a factor of ϵ, then we could assume that the same result can be achieved using just the standard NS run with more live points. In particular, the number of live points would have to be increased by $\epsilon^{2}$, which would also increase the computational complexity by a factor of $\epsilon^{2}$, i.e., $T_{\epsilon }=\epsilon^{2}T$. In contrast, the post-processing with IFT adds a constant, live point independent computational complexity $T_{\mathrm{IFT}}$ to the original computational effort *T* in order to reduce the error by ϵ, i.e., $T_{\epsilon ,\mathrm{IFT}}=T+T_{\mathrm{IFT}}$. Therefore, in cases where *T* is high, e.g., when the KL is high, or in particular when the evaluation of the likelihood or the sampling from the restricted prior requires a lot of time, it makes sense to consider the inference of the likelihood instead of the addition of further live points.

If we examine the reconstructed likelihood–prior–volume curves shown in Section 3, we find that the prior volumes for the spike-and-slab likelihood show an abrupt change in the prior volume in the interval lnX∈[−20,−30]. In fact, this quick change in the prior volume corresponds to small slope of the likelihood–prior–volume curve in this interval, which would need to be modelled by a large value of τ. Looking at the prior generative model shown in Section 2.2, this particular case is difficult to model, as the correlated field would need to be a straight line with a peak in this interval, which it is hardly forced to by the relatively few data points given for $n_{\mathrm{live}}=2$ or $n_{\mathrm{live}}=10$. We expect this to look better for examples that have several changes in the slope of the curve. In any case, looking at the results in Table 2 and Table 4, we see that the reconstruction works quite well during validation for the Gaussian and spike-and-slab examples. However, it is noticeable that the benefit of using post-processing decreases as the number of live points increases. This is expected because the likelihood–prior–volume function generated by NS itself becomes smoother as the number of live points increases. Nevertheless, we can see an increase in the accuracy of the result for the log-evidence up to one hundred live points in terms of a decreased standard deviation. When the number of live points increases even further, we find that the final $\sigma_{\delta }$, defined by the minimum distance between two adjacent nested likelihood contours, becomes very small. This can especially be seen in the case of the spike-and-slab likelihood for one thousand live points, where $\sigma_{\delta }=2.26\times 10^{-10}$. As a result, the reconstruction using the Gaussian approximation of the δ-function becomes numerically unstable, in the sense of a high reduced $\chi^{2}$ value between the data and the reconstruction, allowing the reconstruction to be performed with fewer sample chains and obtaining a worse estimate of the log evidence. Therefore, the likelihood–prior–volume function inference proposed here is not applicable to a large number of live points. Instead, it could be used to improve the log-evidence calculation in scenarios where only a small number of live points are feasible, e.g., due to high LRPS costs. The applicability with respect to the reduced $\chi^{2}$ and the inherent uncertainty given by the algorithm should therefore be checked by the user.

Thus, this post-processing method leaves some room for future work. First of all, it would be desirable to apply it to a problem with a large number of live points. Two different approaches could be considered. First, one could split the dataset into parts and thus generate NS datasets with a smaller number of live points and thus a larger distance between adjacent nested likelihood contours [11]. Second, one could think of ways to avoid the Gaussian approximation of the δ-function. One alternative approach that does not require the Gaussian approximation of the δ-function is described in the Appendix C. Using this approach for VI is left for future work. Moreover, one could think of further assumptions besides the smoothness assumption and include them in the inference to allow for a wider range of likelihoods, such as non-smooth ones or a specific set of likelihoods.

In conclusion, we have presented a post-processing step for NS that uses a smoothness assumption to infer the likelihood–prior–volume function, providing an estimate of the inherent uncertainty in the reconstruction due to the stochastic approach. This aims to reduce the statistical error in the evidence computation introduced by the unknown prior volumes at each iteration step. Since this post-processing can deal with a varying number of live points, it is applicable to advanced NS methods such as dynamic NS. Finally, some work needs to be done to apply it to problems with a larger number of live points. However, the method presented here should provide a first approach to improving prior volume estimation, which remains one of the main challenges in NS.

## Figures and Tables

**Table 1:** Hyperparameters for correlated field samples shown in Figure 2. The reference parameters are denoted by an index **r**. The other indices correspond to the labels of the sub-figures (**a**, **b**, **c**). Modified hyperparameter means with respect to the reference field are marked in blue.

	$\eta_{r}$	$m_{r}$	$a_{r}$	$\eta_{a}$	$m_{a}$	$a_{a}$	$\eta_{b}$	$m_{b}$	$a_{b}$	$\eta_{c}$	$m_{c}$	$a_{c}$
Mean	0.5	−6	1.0	0.5	−6	3.0	0.5	−2	1.0	10.0	−6	1.0
Std	0.5	$10^{-16}$	0.5	0.5	$10^{-16}$	0.5	0.5	$10^{-16}$	0.5	0.5	$10^{-16}$	0.5

**Table 2:** Inferred (IFT) and NS (NS stat: statistical, NS det: deterministic) results for the computed Gaussian log-evidence, represented by the mean and the according standard deviation for $n_{\mathrm{live}}\in \left\{2,10,1000\right\}$ given *K* sample chains. The ground truth is lnZ=−30.87. The histograms of the distributions of the evidences are shown in Figure 4.

$n_{\mathrm{live}}$	*K*	Mean			Standard Deviation	
		IFT	NS Stat	NS Det	IFT	NS Stat
2	120	−28.51	−25.11	−20.90	2.98	3.23
10	120	−30.56	−30.44	−29.37	0.24	1.6
1000	120	−31.01	−30.52	−30.51	0.61	0.17

**Table 3:** Parameters for the spike-and-slab likelihood described in Equation (29).

Parameter	Meaning	Value
*C*	number of dimensions	10
*a*	relative weight of Gaussians	0.5
$\sigma_{1}$	std of Gaussian weighted by *a*	0.1
$\sigma_{2}$	std of Gaussian weighted by (1−a)	0.02

**Table 4:** Inferred (IFT) and NS (NS stat: statistical, NS det: deterministic) results for the computed spike-and-slab log-evidence, represented by the mean and the according standard deviation for $n_{\mathrm{live}}\in \left\{2,10,1000\right\}$ given *K* sample chains. The ground truth is lnZ=−15.47. The histograms of the distributions of the evidences are shown in Figure 5.

$n_{\mathrm{live}}$	*K*	Mean			Standard Deviation	
		IFT	NS Stat	NS Det	IFT	NS Stat
2	40	−17.26	−13.00	−11.21	1.67	1.91
10	40	−16.03	−14.62	−14.01	0.78	0.80
1000	20	−18.35	−15.49	−15.50	0.01	0.10

## Data Availability

Data sharing not applicable. The corresponding implementation is public and available at https://gitlab.mpcdf.mpg.de/ift/public/iftns (accessed on 24 October 2024).

## Abbreviations

The following abbreviations are used in this manuscript:

geoVI	geometric variational inference
IFT	information field theory
KL	Kullback-Leibler divergence
LRPS	likelihood restricted prior sampling
MCMC	Markov Chain Monte Carlo
NIFTy	Numerical Information Field TheorY
NS	nested sampling
NS-SMC	nested sampling via sequential Monte Carlo
VI	variational inference

## Appendix A. Reparametrisation

The aim of the reparametrisation is to map the likelihood–prior–volume function such that the correlated field describing the log-normal process is constant in the Gaussian case and non-Gaussianity is modelled by structures in the correlated field (Section 2.2). Below, we show that the reparametrisation in Equation (18) leads to this condition. For the Gaussian case (Equation (23)), we have

(A1) $$\begin{array}{c} \frac{d\ln L}{d\ln X}=\frac{d}{d\ln X}\left(-\frac{X^{2/C}}{2\sigma^{2}}\right)=\frac{2}{C}\ln L. \end{array}$$

For the Gaussian case, we know $\ln L_{\max}=0$ analytically. If we substitute this into Equation (18) we find the desired condition,

(A2) $$\begin{array}{c} \frac{df(\ln L)}{d\ln X}=-\frac{2}{C}=-e^{-\tau (\ln X)}, \end{array}$$

leading to τ(lnX)=const. It can be seen that the corresponding reparameterisation is given by the following equation

(A3) $$\begin{array}{c} f\left(\ln L\right)=-\ln\left(\ln\frac{L_{\max}}{L}\right). \end{array}$$

## Appendix B. Maximal Likelihood Calculation

We calculate the maximum likelihood given the data on the likelihood dead contours ${\vec{d}}_{L}$. In [35] the maximum of the Shannon entropy $I_{\max}=\max\ln\left(\frac{P(\theta |d)}{P(\theta )}\right)$ is calculated via,

(A4) $$\begin{array}{c} I_{\max}=D_{\mathrm{KL}}+\frac{C}{2}, \end{array}$$

where *C* is the dimension of the problem. This gives the maximum log-likelihood $\ln L_{\max}$,

(A5) $$\begin{array}{cc} \ln\left(\frac{L_{\max}}{Z}\right) & =\int d\theta P\left(\theta |d\right)\ln\left(\frac{L}{Z}\right)+\frac{C}{2} \end{array}$$

(A6) $$\begin{array}{cc} \to \ln L_{\max} & ={\langle \ln L\rangle }_{P(\theta |d)}+C/2. \end{array}$$

This can be approximated using the data on the likelihood contours and taking the deterministic NS information on the prior volumes $\left\{d_{X,i}\right\}$ according to Equation (8),

(A7) $$\begin{array}{cc} \ln L_{\max} & =\int d\theta \frac{P(d|\theta )P(\theta )}{Z}\ln L+\frac{C}{2} \end{array}$$

(A8) $$\begin{array}{cc} \; & \approx \sum_{i=1}^{n_{\mathrm{iter}}}\frac{1}{2}\frac{d_{L,i}\left(d_{X,i-1}-d_{X,i+1}\right)}{Z_{d}}\ln d_{L,i}+\frac{C}{2}. \end{array}$$

Here, $Z_{d}$ is the evidence calculated according to Equation (3), with $\left\{d_{L,i}\right\}$ as the likelihood information and $\left\{d_{X,i}\right\}$ as the prior volume information.

## Appendix C. Inference with Gaussian Approximation

In the following, we want to work out an exact inference algorithm that is not based on a Gaussian approximation of the δ-function. This approach has its difficulties due to the calculation of the Fisher metric, as we will see below, and therefore cannot be used for geoVI. The approach discussed in the main part of this paper focuses on the joint reconstruction of the likelihood–prior–volume function and the prior volumes. The approach presented here solely reconstructs the likelihood–prior–volume function, given the data on the likelihood contours. The corresponding prior volumes for each likelihood contour can then be computed by inversion of this function. Again, we incorporate the smoothness assumption into the inference, but this time using a different relationship between the likelihood–prior–volume function and the correlated field,

(A9) $$\begin{array}{c} \frac{df(\ln L)}{d\ln X}=-e^{\tau (f(\ln L))}. \end{array}$$

The solution for the prior volumes given the likelihood information is,

(A10) $$\begin{array}{c} X\left(f\left(\ln L\right)|\tau \right)=\exp\left(\ln X_{0}-\int_{f(\ln L_{0})}^{f(\ln L)}e^{-\tau (y)}dy\right)=\exp\left(-\int_{f(\ln L_{0})}^{f(\ln L)}e^{-\tau (y)}dy\right), \end{array}$$

with $X_{0}=1$. The prior model is then given by P(τ), and our goal is to determine the likelihood model, denoted by $P(f\left(\ln{\vec{d}}_{L}\right)|\tau )$, which can be rewritten by marginalising over $\vec{t}$,

(A11) $$\begin{array}{c} P\left(f\left(\ln{\vec{d}}_{L}\right)|\tau \right)=\prod_{i=1}^{n_{\mathrm{iter}}}\int dt_{i}\;P\left(f\left(\ln d_{L,i}\right),\vec{t}|\tau \right)=\prod_{i=1}^{n_{\mathrm{iter}}}\int dt_{i}\;P\left(t_{i}\right)\;P\left(f\left(\ln d_{L,i}\right),\vec{t}|\tau \right). \end{array}$$

Here, the contraction factors $\vec{t}$ are beta distributed, i.e., $P\left(t_{i}\right)=\mathrm{Beta}\left(t_{i}|1,n_{\mathrm{live}}\right)$. The tricky part is to rewrite $P(f\left(\ln d_{L,i}\right),\vec{t}|\tau )$ such that we can integrate over $t_{i}$. Under the assumption that we can find a unique solution for $f(\ln d_{L,i})$ with $i=1,..,n_{\mathrm{iter}}$ for each of the following $n_{\mathrm{iter}}$ equations using Equation (A10),

(A12) $$\begin{array}{c} X\left(f\left(\ln d_{L,i}\right)|\tau \right)=\prod_{j=1}^{i}t_{j}\;\;\;\mathrm{for}\;i=1,...,n_{\mathrm{iter}} \end{array}$$

the distribution $P(f\left(\ln d_{L,i}\right)|\tau ,\vec{t})$ is defined via

(A13) $$\begin{array}{c} P\left(f\left(\ln d_{L,i}\right)|\tau ,\vec{t}\right)=\delta \left(X\left(f\left(\ln d_{L,i}\right)|\tau \right)-\prod_{j=1}^{i}t_{j}\right)\;|\frac{\partial X(f(\ln L)|\tau )}{\partial f(\ln L)}{|}_{f\left(\ln L\right)=f\left(\ln d_{L,i}\right)}. \end{array}$$

In the following, we will denote $|\partial_{\tau }{X|}_{d_{L,i}}=|\frac{\partial X(f(\ln L)|\tau )}{\partial f(\ln L)}{|}_{f\left(\ln L\right)=f\left(\ln d_{L,i}\right)}$ and $X_{i}=X\left(f\left(\ln d_{L,i}\right)|\tau \right)$, for the matter of brevity of the equations. With the definition of $t_{0}:=1$, we can further rewrite Equation (A13) to,

(A14) $$\begin{array}{cc} P(f\left(\ln d_{L,i}\right)|\tau ,\vec{t}) & =\delta \left(t_{i}-\frac{X_{i}}{\prod_{j=0}^{i-1}t_{j}}\right)\;{\left|\partial_{\tau }X\right|}_{d_{L,i}}\;\prod_{j=0}^{i-1}\frac{1}{t_{j}}, \end{array}$$

(A15) $$\begin{array}{cc} \; & \;\;\;\;\;\;=\delta \left(t_{i}-\frac{X_{i}}{X_{i-1}}\right)\;{\left|\partial_{\tau }X\right|}_{d_{L,i}}\;\prod_{j=0}^{i-1}\frac{1}{t_{j}}, \end{array}$$

Using this and Equation (A11), we can write down the overall reconstruction likelihood for this approach:

(A16) $$\begin{array}{cc} P(f\left(\ln{\vec{d}}_{L}\right)|\tau ) & =\prod_{i=1}^{n_{\mathrm{iter}}}\int dt_{i}P\left(t_{i}\right)\delta \left(t_{i}-\frac{X_{i}}{X_{i-1}}\right)\;{\left|\partial_{\tau }X\right|}_{d_{L,i}}\;\prod_{j=0}^{i-1}\frac{1}{t_{j}} \end{array}$$

(A17) $$\begin{array}{cc} \; & \;\;\;=\prod_{i=1}^{n_{\mathrm{iter}}}\mathrm{Beta}\left(\frac{X_{i}}{X_{i-1}}|1,n_{\mathrm{live}}\right)\frac{1}{X_{i-1}}{\left|\partial_{\tau }X\right|}_{d_{L,i}} \end{array}$$

(A18) $$\begin{array}{cc} \; & =\prod_{i=1}^{n_{\mathrm{iter}}}n_{\mathrm{live},i}\frac{X_{i}^{n_{\mathrm{live},i}-1}}{X_{i-1}^{n_{\mathrm{live},i}}}\;{\left|\partial_{\tau }X\right|}_{d_{L,i}} \end{array}$$

So far, the likelihood model and the prior model can be used for a maximum a posteriori approximation of f(lnL). For variational inference (VI), however, the Fisher metric is required,

(A19) $$\begin{array}{cc} {\left(I_{F}\right)}_{k,l}=\int df\left(\ln d_{L,0}\right)...\int df\left(\ln d_{L,n_{\mathrm{iter}}}\right)\; & \frac{\partial }{\tau_{k}}\left(-\ln\left(P\left(f\left(\ln{\vec{d}}_{L}\right)|\tau \right)\right)\right) \end{array}$$

(A20) $$\begin{array}{cc} \; & \times \frac{\partial }{\tau_{l}}\left(-\ln\left(P\left(f\left(\ln{\vec{d}}_{L}\right)|\tau \right)\right)\right). \end{array}$$

Due to the complexity and length of the calculations involved in this integration and the difficulties of its implementation, we have opted to use the Gaussian approximation and reserve this approach for future work.
